# Surgical Treatment of 55 Patients with Pressure Ulcers at the Department of Plastic and Reconstructive Surgery Kosovo during the Period 2000–2010: A Retrospective Study

**DOI:** 10.1155/2013/129692

**Published:** 2013-02-20

**Authors:** Shkelzen B. Duci, Hysni M. Arifi, Mimoza E. Selmani, Agon Y. Mekaj, Musli M. Gashi, Zejn A. Buja, Vildane H. Ismajli, Adem N. Kllokoqi, Enver T. Hoxha

**Affiliations:** ^1^Department of Plastic and Reconstructive Surgery, University Clinical Center of Kosovo, Pristina, Kosovo; ^2^Dentistry Faculty, University Clinical Center of Kosovo, Pristina, Kosovo; ^3^Department of Neurosurgery, University Clinical Center of Kosovo, Pristina, Kosovo; ^4^Department of Emergency Center Kosovo, Pristina, Kosovo

## Abstract

*Objective*. The objective of this study is to determine the incidence of PUs, the distribution of PUs, common injuries contributing to the occurrence of PUs in patients admitted to the Department of Plastic and Reconstructive Surgery Kosovo for surgical interventions of PUs, localization of PUs in body, the topical treatment of pressure ulcers before surgical intervention, the methods of surgical interventions, number of surgical interventions, duration of treatment, complications, and mortality. *Materials and Methods*. This study includes 55 patients with PUs treated surgically in 2000–2010 period in the Department of Plastic and Reconstructive Surgery Kosovo. The data were collected and analyzed from the archives and protocols of the University Clinical Center of Kosovo. Data processing was done with the statistical package In Stat 3. From statistical parameters arithmetic median and standard deviation were calculated. Data testing is done with *χ* ^2^-test and the difference is significant if *P* < 0.05. *Conclusion*. Despite preventive measures against PUs, the incidence of Pus remains high.

## 1. Introduction

Pressure ulcers (PUs) are defined as localized injury to the skin or underlying tissue usually over bony prominence, as a result of pressure, or pressure in combination with shear or friction [1, 2]. Pressure ulcers are almost a serious, secondary complication of spinal cord injury that has the potential to interfere with physical, psychological, and social well being and to impact overall quality of life [3]. PUs are classified by the level of visible tissue damage, where stage I PUs exhibit nonblanchable erythematic (i.e., redness) on intact skin, stage II PUs are partial thickness ulcers, and stages III and IV ulcers involve full-thickness damage [4]. They are believed to occur from combination of extrinsic forces such as pressure, shear, and friction and intrinsic factors such as age, malnourishment, and consciousness level that influence a person's tissue tolerance [5, 6]. Previous studies have identified the following factors as increasing the likelihood of developing a pressure ulcer: immobility, admission to the ICU, malnutrition, incontinence, hypoalbuminemia, spinal cord injury, stroke, reduced level of consciousness, fractures and/or major orthopedic procedure, advanced age, trauma, decreased perfusion, poor wound healing, inadequate nursing care, and chronic illness [7–10]. 

Contributing risk factors increase the patient's susceptibility to a complex etiology that causes PUs [11]. 

Debridement of pressure sores often results in extensive soft tissue defects that cannot be closed primarily and are further associated with increased risk of flap ischemia, wound dehiscence, and deep infection [12]. Numeral surgical methods have been used to correct these defects, including skin grafting [12, 13], local flaps [12, 14], muscle flaps [12, 15], and free flaps [12, 16].

## 2. Objective

The objective of this study is to determine the incidence of PUs in our population, the distribution of PUs, common injuries contributing to the occurrence of PUs in patients admitted to the Department of Plastic and Reconstructive Surgery Kosovo for surgical interventions of PUs, localization of PUs in the body, the topical treatment of pressure ulcers before surgical intervention, the methods of surgical interventions, number of surgical interventions, duration of treatment, complications and mortality.

## 3. Materials and Methods

This is a retrospective study that included 55 patients with 72 defects caused from PUs treated surgically in 2000–2010 period in the Department of Plastic and Reconstructive Surgery Kosovo. The data were collected and analyzed from the archives and protocols of the University Clinical Center of Kosovo. This research project was approved by the Regulation and Ethical Standards Commission. In this study we included patients with PUs stages III and IV who underwent surgical interventions, and we excluded the patients who underwent topical treatments of small wounds without surgical intervention. Data processing was done with the statistical package In Stat 3. From statistical parameters arithmetic median and standard deviation were calculated. Data testing is done with *χ* ^2^-test and the difference is significant if *P* < 0.05. 

## 4. Results 

**Figure 1:**
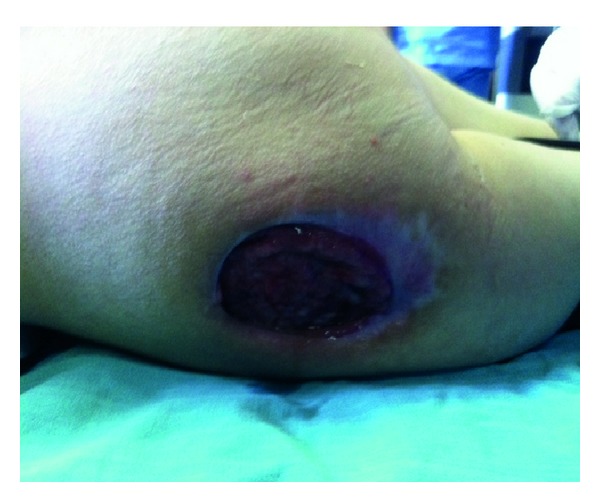
Pressure ulcer in 16-year-old patient with spinal cord injury in trochanteric region after topically treatment of the wound.

**Figure 2:**
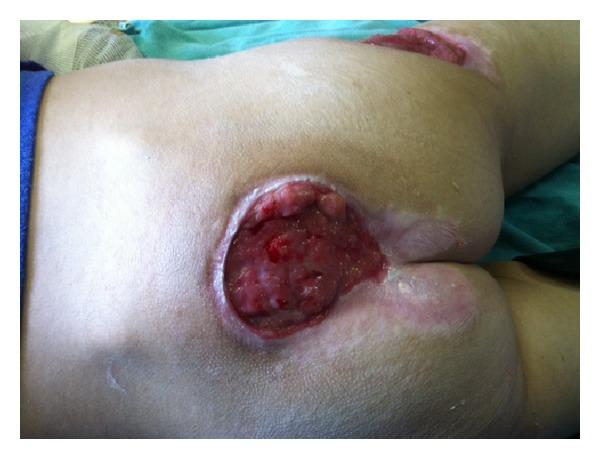
Pressure ulcer in the same patient in sacral region after topical treatment.

**Figure 3:**
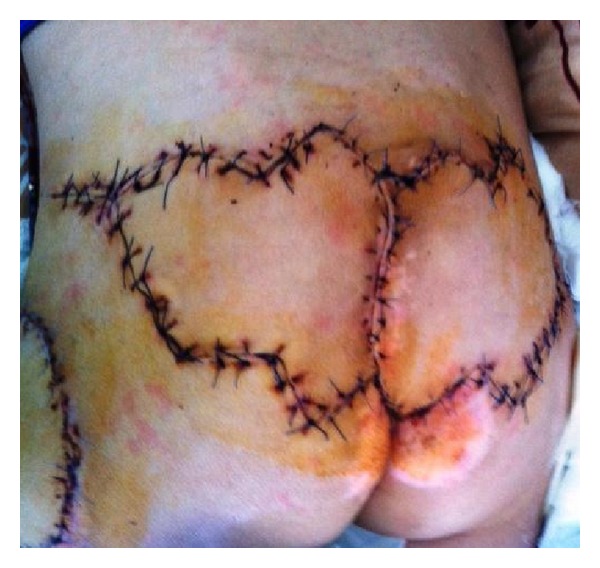
Sacral defect covered with two musculocutaneous advancement flaps.

**Figure 4:**
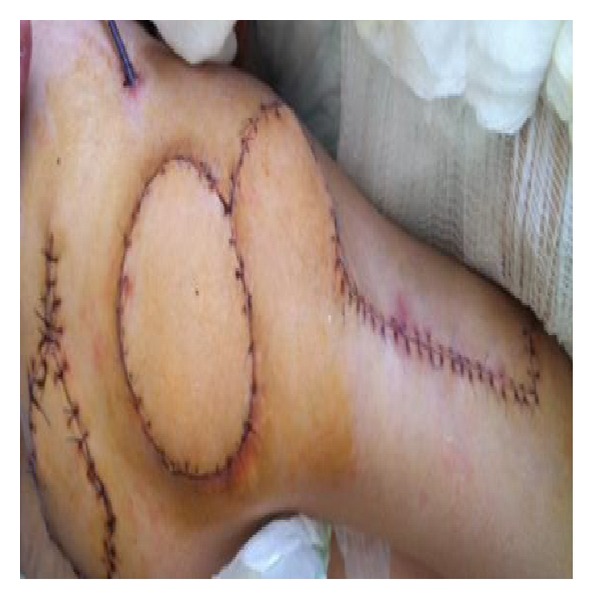
Right trochanteric defect covered with TFL flap.

In this study PUs were predominant in male patients with 42 cases or 76.3% with only 13 cases or 23.6% in female patients (Table 1). The incidence of pressure ulcers was noted to be higher in the age group 30–39 with 20 cases or 36.3% followed by children where the children are considered up to the age 19 years old by WHO with 10 cases (18.1%), 20–29 years 9 cases (16.3%), 40–49 years 5 cases (9%), 50–59 years 5 cases (9%), 60–69 years 2 cases (3.6%), and over 70 years 4 cases (7.2%). The average age of patients was 34.8 years (Table 1). We found that patients with spinal cord injuries had the highest incidence of PUs with 48 cases, followed by patients with cerebral injuries 3 cases, orthopedic traumatic injuries with 3 cases, and 1 case with congenital anomaly of spinal cord. From 48 patients with spinal cord injuries 37 patients or 77% were male and 11 patients or 22.9% were female; from 3 patients with cerebral injuries 2 patients were male and 1 patient were female; from 3 patients with orthopedic traumatic injuries 2 patients were male and 1 patient was female, in addition to 1 case (male) with congenital anomaly of spinal cord. Distribution of PUs in years had the following features: 2002 and 2003 are the years where the incidence of PUs was the highest with 7 cases (12.7%); 2005 with 6 cases (10.9%); 2001, 2006, 2007, and 2008 with 5 cases (9%); 2004, 2009, and 2010 with 4 cases (7.2%), and 2000 with only 3 cases (5.5%). In terms of body localization of pressure ulcers, the most frequent localization was the sacral region with 41 cases (74.5%). Other localizations had the following distribution: the trochanteric region with 12 cases (21.8%), ischia region 11 cases (20%), femoral region 3 cases (5.4%), occipital region 2 cases (3.6%), malleolar region 2 cases (3.6%), and calcaneal region 1 case (1.8%) (Table 2). Topical treatment of the wounds before surgical intervention was mostly performed with wound dressing with povidone iodine solution and silver sulfadiazine ointment. For coverage of defects caused by PUs 20 musculocutaneous flaps, including V-Y flaps and transposition (36.3%) usually to cover defects over sacral region and 18 cutaneous local flaps (32.7%); 12 small defects are closed with direct closure (21.8%); in 12 cases cutaneous grafts (21.8%) were used, and in 10 cases fasciocutaneous flap of tensor fascia lata for reconstruction of trochanteric region (18.1%) (Figures 1, 2, 3, and 4) (Table 2). Forty-five cases underwent surgical intervention only once, while 7 cases had two surgical interventions. Three cases required surgical intervention because of partial necrosis of the flaps and dehiscence (Table 3). Duration of treatment ranged from 8 to 178 days. The mean hospitalization was 63.6 days. Four cases were complicated with necrotizing fasciitis and sepsis from which two cases died (3.6%) [3].

## 5. Discussion

PUs are serious health problem [17, 18]. They cause pain and distress in the affected individuals. Their treatment is very costly for the health care system and the society [17, 19]. In this study PUs were predominant in male patients with 42 cases or 76.3% with only 13 cases or 23.6% in female patients (Table 1). The highest incidence of pressure ulcers was noted in the age group from 30 to 39 years old with 20 cases or 36.3% followed by children (children are considered up to the age 19 years old by WHO) with 10 cases (18.1%), 20–29 years 9 cases (16.3%), 40–49 years 5 cases (9%), 50–59 years 5 cases (9%), 60–69 years 2 cases (3.6%), and over 70 years 4 cases (7.2%). We found that PUs had the highest incidence in patients with spinal cord injuries with 48 cases, followed by patients with cerebral injuries 3 cases, orthopedic traumatic injuries with 3 cases, and 1 case with congenital anomaly of spinal cord. Out of 48 patients with spinal cord injuries 37 or 77% of them were male and 11 patients or 22.9% were female. Two out of 3 patients with cerebral injuries were male and only one patient was female (all cases are associated with coma after severe cerebral injury). Two out of 3 patients with orthopedic traumatic injuries were male (1 with amputation of right extremities and another associated with fracture of pelvic bone) and 1 patient was female (with septic arthritis associated with head necrosis of femoral bone), with 1 case with congenital anomaly of spinal cord (myelomeningocele). The most important finding which was not noted in other studies is the predominance of the young age 31–40 years with 20 cases; the average age of patients in our study is 34.8 years. A similar study of 60 patients with PUs by Schiffman et al. reported that average age of patients in their study was 73.1 [20]. This high incidence of PUs was noted in patients with spinal cord injuries and also the predominance of young age in our country probably results from a couple of factors. After 1999, our country emerged from the war and many deadly weapons still remain in the hands of our citizens; therefore spinal cord injuries by firearms were very frequent. Detailed history and physical examination showed that 18 cases or 32.7% of these patients had spinal cord injuries from firearms. Traffic accidents were the second most common cause of spinal cord injuries. Distribution of PUs in years had the following features: 2002 and 2003 are the years where the incidence of PUs was the highest with 7 cases (12.7%); 2005 with 6 cases (10.9%); 2001, 2006, 2007 and 2008 with 5 cases (9%); 2004, 2009, and 2010 with 4 cases (7.2%); and 2000 with only 3 cases (5.5%). Body localization of pressure ulcers has the following features: the most frequent localization was the sacral region in 41 cases (74.5%). Other localizations had the following distributions: the trochanteric region with 12 cases (21.8%), ischia region 11 cases (20%), femoral region 3 cases (5.4%), occipital region 2 cases (3.6%), maleolar region 2 cases (3.6%), and calcaneal region 1 case (1.8%). In other similar studies conducted by Nogueira et al. in 46 patients with spinal cord injuries the sacral region had the most frequent localization with 17 cases, followed by heel region with 8 cases and gluteus region with 5 cases [21]. Topical treatment of the wounds before surgical intervention usually is done with wound dressing of povidon iodine solution and sulfadiazine ointment. Our department is not fully equipped with modern technology for treatment of PUs such as wound closure device of vacuum-assisted closure techniques, topical application of growth factors, and health care products designed for local pressure distribution. Therefore depending on the local status of the wound we determine which of the following dressings (povidon iodine solution and sulfadiazine ointment) will be used for treatment of these wounds. Also some patients are followed in the operating room for debridement of necrotic tissue before the surgical intervention in order to prepare pressure ulcers for surgical closure. For coverage of defects due to PUs 20 musculocutaneous flaps, included V-Y flaps and transposition (36.3%) usually to cover defects in the sacral region, 18 cutaneous local flaps (32.7%) are used; 12 small defects are closed with direct closure (21.8%), in 12 cases cutaneous grafts (21.8%) were used, and in 10 cases fasciocutaneous flap of tensor fascia lata for reconstruction of trochanteric region (18.1%). In pressure ulcers over sacral and trochanteric regions (30 cases) 20 musculocutaneous and 10 fasciocutaneous flaps were used. The operative treatment was done with complete pressure ulcer excision, removal of dead and necrotic tissue, lavage with hydrogen peroxide, and achievement of hemostasis. Bone debridement and contouring are performed with rongeurs and rasps and the final procedure was placement of drains. In 5 cases after reconstruction of defects in sacral region with transposition flaps cutaneous grafts were used to cover this defects together with flap. From 18 cutaneous local flaps 10 flaps were transposition, 6 rotation, and 2 V-Y flaps and were used to cover small defects caused by pressure ulcer. In 6 cases together with transposition local cutaneous flaps were used and cutaneous grafts to cover this defects while in 6 cases with reconstruction of defects with rotation flaps also cutaneous grafts were used. In 12 cases with small defect were closed with direct closure of the wound. Forty-five cases underwent surgical intervention only one time and they do not have postoperative complication of the flaps while 7 cases had two surgical interventions from which 5 cases after direct closure because of dehiscence of the wounds and 2 after reconstruction of defects with cutaneous local flaps because of partial necrosis of flaps, and 3 cases had three surgical interventions from which 2 after direct closure because dehiscence of wound and 1 after reconstruction of defects with cutaneous local flaps because of partial necrosis of flap. Another important finding in our study, which is not noted in other studies, is the hospitalization of patients in our department ranged from 8 to 178 days. The mean hospitalization was 63.6 days. Alderden et al. in their study in 87 patients with PUs found that the mean of hospitalization was 37 days [22]. This difference in our study compared with other studies done for PUs probably results from some reasons. Our department is the only department in our country that treats the patients with PUs from debridement of the wound, local treatment and preparation of patients for surgical interventions and their postoperative care. Thereupon the treatment of patients with PUs in our department had a long stay hospitalization. Four cases are complicated with necrotizing fasciitis and sepsis from which two cases died (3.6%). Detailed history taking and physical examination showed that in most of these patients infection would spread from sacral and trochanteric region down towards the femoral region all the way to the knee level and was complicated with necrotizing fasciitis and sepsis. Major factor contributing to high mortality in these patients is considered delayed referral by primary care services. If patients are referred late, mortality remains significantly high (despite radical debridement of wounds and systemic antibiotic therapy two cases died and two cases survive the disease in our study). 

## 6. Conclusion

Management of pressure ulcers lies mainly in its prevention. Despite preventive measures against PUs, the incidence of PUs remains high. In our study population the incidence of PUs was very high in patients with spinal cord injuries caused by firearms, where vast majority of injuries were noted in the 2000–2010 period. The average age of patients with PUs was 34.6. In 1999, our country emerged from war; however a lot of deadly weapons still remain illegally in the hands of our citizens. As a consequence the number of spinal cord and other injuries causing pressure ulcers is much higher as compared to the other neighboring countries. Once pressure ulcers develop their course, there are variety of treatment modalities. Topical treatment of the wounds before surgical intervention usually is done with wound dressing of povidon iodine solution and sulfadiazine ointment. Our department, in cooperation with the Ministry of Health, should make effort to secure other modern devices for treatment of PUs such as vacuum-assisted closure techniques, topical application of growth factors, and health care products designed for local pressure distribution and to organize special training courses for use of these devices. We used musculocutaneous flaps to cover sacral defects, whereas fasciocutaneous flaps of TFL were used in trochanteric region. The use of cutaneous local flaps for coverage of defects and direct closure in 10 patients was accompanied with partial necrosis of flaps and dehiscence of the wound. Based on this study we can conclude that the use of cutaneous local flaps and direct closure is not appropriate option for these defects. Subsequently flaps should routinely be utilized for these complicated defects. Four cases are complicated with necrotizing fasciitis and sepsis from which two cases died (3.6%). 

Spread of infection was noted from sacral and trochanteric regions towards the femoral region all the way down to the knee level and was complicated with necrotizing fasciitis and sepsis. Major factor contributing to high mortality in this patient population is delayed referral by primary care services. As a consequence we need to organize special training programs for practitioners in primary care services through which they will be familiarized with PU pathology, treatment, and complications. The involvement of families and other medical professionals is essential for successful treatment of PUs.

## Figures and Tables

**Table 1:** General characteristics of patients with PUs.

	No (*n* = 55)	%
Gender*		
Male	42	76.3
Female	13	23.6
Age groups (years)		
Children	10	18.1
20–29	9	16.3
30–39	20	36.3
40–49	5	9
50–59	5	9
60–69	2	3.6
70+	4	7.2
Mean ± SD	34.8 ± 10.0	
Range	11–79 years	

*Significant by gender (*χ* ^2^-test = 15.3, *P* < 0.001).

**Table 2:** Localization of PUs and surgical methods used for treatment of defects caused by Pus.

	No (*n* = 72)	%
Localization by the regions		
Sacral region	41	74.5
Trochanteric	12	21.8
Ischia	11	20
Femoral	3	5.4
Occipital	2	3.6
Malleolar	2	3.6
Calcaneal	1	1.8
Surgical methods		
Musculocutaneous flaps	20	36.3
Cutaneous local flaps	18	32.7
Direct closure	12	21.8
Cutaneous grafts	12	21.8
Fasciocutaneous flaps TFL*	10	18.1

*TFL-tensor fascia lata.

**Table 3:** Duration of hospitalization, number of surgical interventions, complications, and morbidity in patients with pressure ulcers.

	No (*n* = 55)	%
Number of surgical interventions		
1 time	45	81.8
2 times	7	12.7
3 times	3	5.4
Complications		
Necrotising fasciitis	4	7.2
Morbidity (sepsis)	2	3.6
Duration of hospitalization (days)		
Range	8–178 days	
Mean ± SD	63.6 ± 10.0	8–178 days
